# Effect of Cross-Linking Density on Non-Linear Viscoelasticity of Vulcanized SBR: A MD Simulation and Experimental Study

**DOI:** 10.3390/ijms24129970

**Published:** 2023-06-09

**Authors:** Tian Yan, Ke-Jian Wang, Xiu-Ying Zhao, Yang-Yang Gao

**Affiliations:** 1 College of Mechanical and Electrical Engineering, Beijing University of Chemical Technology, Beijing 100029, China; 2 Key Laboratory of Beijing City on Preparation and Processing of Novel Polymer Materials, Beijing University of Chemical Technology, Beijing 100029, China

**Keywords:** vulcanized SBR, non-linear viscoelasticity, Payne effect, MD simulations

## Abstract

In recent years, there has been a growing interest in changes in dynamic mechanical properties of mixed rubber during dynamic shear, yet the influence of vulcanized characteristics on the dynamic shear behavior of vulcanized rubber, particularly the effect of cross-linking density, has received little attention. This study focuses on styrene–butadiene rubber (SBR) and aims to investigate the impact of different cross-linking densities (*D*_c_) on dynamic shear behavior using molecular dynamics (MD) simulations. The results reveal a remarkable Payne effect, where the storage modulus experiences a significant drop when the strain amplitude (γ_0_) exceeds 0.1, which can be attributed to the fracture of the polymer bond and the decrease in the molecular chain’s flexibility. The influence of various *D*_c_ values mainly resides at the level of molecular aggregation in the system, where higher *D*_c_ values impede molecular chain motion and lead to an increase in the storage modulus of SBR. The MD simulation results are verified through comparisons with existing literature.

## 1. Introduction

Rubber is a cost-effective viscoelastic material with exceptional elasticity performance that finds extensive applications in automobiles, aerospace, construction, and medicine [1,2,3]. Since rubber products are usually subjected to alternating loads [4], the processing technology has a significant impact on their mechanical properties, particularly their dynamic mechanical properties. Studying the influence of processing technology on rubber’s dynamic mechanical properties is crucial to enhancing its mechanical performance.

Pure rubber cannot meet practical mechanical performance requirements. Thus, the most common method to improve rubber mechanics is adding microfillers. Silicon dioxide particles and carbon black (CB) are the most common microfillers used to improve rubber mechanics. Jong et al. [5,6] showed that adding silica fillers significantly improves natural rubber’s tensile properties, while Hait et al. [7] demonstrated the enhanced mechanical effect of adding CB to polybutadiene rubber (BR). Lin et al. [8] prepared CB-filled powdered natural rubber (P (NR/N234)) by the method of latex–CB coagulation technology and deeply studied the influence of curing recipes and CB contents on the curing properties, mechanical properties, and dynamic properties, and the results were compared with those of NR/N234 compounds based on traditional dry mixing of bale natural rubber and CB. Robertson and Hardman discussed in detail the mechanism of CB reinforcing the mechanical properties of rubber and the generation mechanism of the Payne effect in the shear process of filling rubbers [9], and there is no doubt that this work is helpful for understanding the reinforcing mechanism and Payne effect of fillers on rubber.

The Payne effect relates to the elastic and storage moduli variation, which typically increase and decrease, respectively, under high shear strain. The phenomenon was first reported by Payne and has since garnered considerable attention. It has been extensively applied in dynamic mechanical analysis for polymer materials under medium-to-high strain. The addition of nanoparticles (NPs) to the mixed rubber has a significant impact on the Payne effect. Shi et al.’s research studied the enhancement mechanism of the Payne effect by using CB to fill natural rubber, and the results indicated the recoverability and hysteresis of the Payne effect, demonstrating the positive impact of high frequencies and low temperatures on enhanced Payne effect. The structural evolution of the filler phase was not the primary influence factor [10]. Similarly, Zhao et al. [11] applied CB as a filler to SBR to create a CB conductive network and studied the Payne effect of the filling system. The results revealed that the polymer matrix next to CB particles can accelerate the reconstruction of the filler–polymer network, thereby enhancing the reversibility of the Payne effect [12,13,14]. CB, along with other fillers, such as spherical [15,16,17] and fibrous [18,19,20], have excellent electrical and thermal conductivity, which significantly affects Payne effect improvement. Experimental research can satisfactorily explain the Payne effect of mixed rubber filling. However, the micromechanism of Payne effect formation remains challenging to explain.

Molecular dynamics (MD) simulation is often preferred to experiments for studying the microscopic dynamic mechanisms of the Payne effect in rubber. MD has several advantages over experiments because it can explore the impact of molecular chain motion and filler–polymer interaction forces, which are typically unknown under experimental conditions. Hong et al. [21] demonstrated the utility of non-equilibrium molecular dynamics (NEMD) in exploring the viscoelastic properties of nanoplate-filled polymer composites as it relates to filler packing fraction, filler–polymer interaction forces, Rouse dynamics, chain constraint, and percolation networks. Gao et al. [22] used hydrodynamic effects, “clog rubber” and “filler network”, to explain how polymer nanocomposites filled with nanorods experience dynamic modulus reinforcement and showed that the Payne effect becomes more pronounced with stronger interface interaction and increased nanorod fraction.

Currently, most research on the Payne effect has focused on mixed rubber, with little research done on vulcanized rubber. Vulcanization plays a critical role in rubber processing, but existing research about the impact of vulcanization characteristics on the Payne effect of rubber is limited. Therefore, it is necessary to investigate the effect of different vulcanization characteristics on the dynamic mechanical properties of rubber. Hou et al. [23] conducted experiments on the Payne effect of polyisoprene rubber vulcanized using thermooxidative aging, and the results revealed that thermooxidative aging significantly affected the vulcanized structure and Payne effect of polyisoprene rubber. Employing a highly efficient vulcanization method to prepare vulcanized polyisoprene rubber will remarkably enhance Payne dissipation, as for its microscopic mechanism, it was not mentioned in this report.

The application scope of vulcanized SBR is wide; therefore, the study of its Payne effect holds theoretical and practical significance. This research aims to utilize molecular dynamics methods to explore the Payne effect of vulcanized SBR in dynamic shear processes, providing new ideas for the study of the dynamic mechanical behavior of sulfurized rubber materials.

## 2. Results and Discussion

### 2.1. Verification of Simulation Correctness

The cross-linking densities (mol/mL) corresponding to each *D*_c_ in MD model obtained via calculation are presented in Table 1. The results of cross-linking densities obtained by experiments are also shown in Table 1.

The small difference between calculations and the experimental results suggests that MD simulation is able to realistically simulate the experiment, thereby demonstrating high reliability in this study.

**Figure 1:**
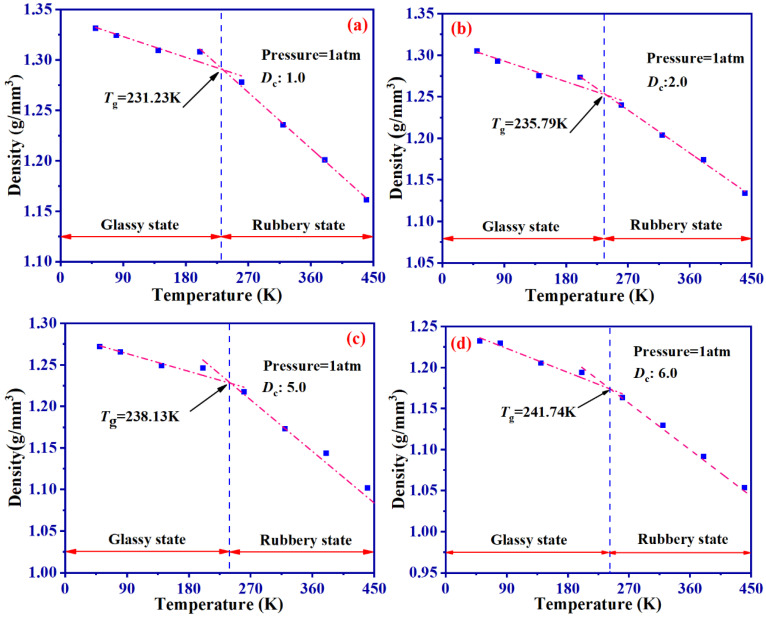

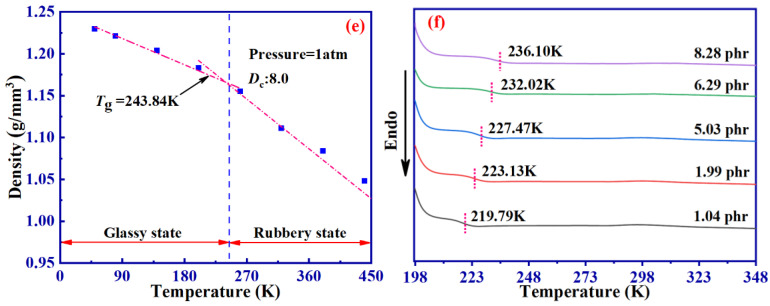
*T*_g_ of different *D*_c_ obtained by MD simulation (**a**–**e**) and DSC (**f**).

In order to prove the accuracy of simulation model, we perform a comparison between *T*_g_ of SBR systems exhibiting varying *D*_c_, utilizing both molecular simulation and experimentation approaches. Figure 1a–e depict the relationships between density and temperature of SBR systems at different *D*_c_ through molecular dynamic simulation. Figure 1f illustrates the *T*_g_ of each sample by experiments. Table 2 enumerates the *T*_g_ for SBR systems of different *D*_c_ acquired through both the experimental and molecular dynamic simulation methods, alongside the corresponding relative deviation.

By comparing the DSC results of different S contents with the *T*_g_ results obtained through simulation (Figure 1), it can be found that the results of MD simulation can predict the *T*_g_ relatively well.

**Figure 2:**
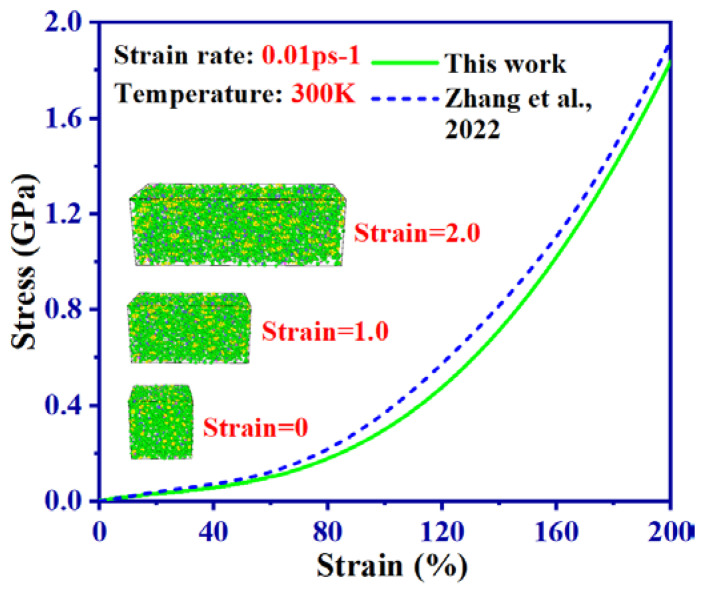
The comparison of the tensile curves of pure SBR at strain rate of 0.01 ps^−1^ and the snapshots at the strains of 0, 1 and 2 with the tensile curves of pure SBR obtained by identical simulation parameters in [24].

The tensile curve of pure SBR at strain rate of 0.01 ps^−1^ is shown in Figure 2, where snapshots captured at strains of 0, 1, and 2 are also provided. Additionally, Figure 2 presents the tensile curve obtained using the identical simulation parameters in reference [24]. It can be found in Figure 2 that the tensile strength of pure SBR is approximately 2 GPa at a strain rate of 0.01 ps^−1^, consistent with the data reported in [24]. The high reliability of the *pcff* force field we selected is thus demonstrated.

### 2.2. The Effects of D_c_ and γ_0_ on Dynamic Mechanical Properties

**Figure 3:**
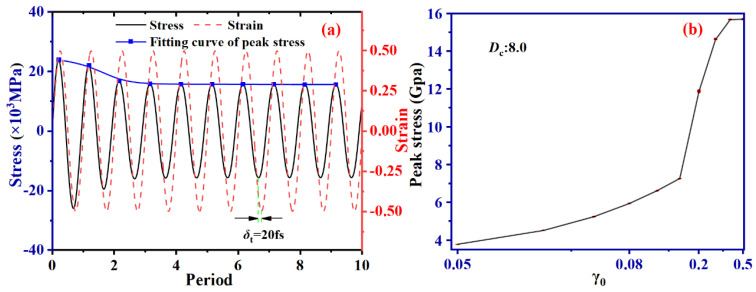
Shear stress−strain curve (**a**) and equilibrium stress peak (**b**) of SBR system with *D*_c_ = 8.0 at γ_0_ = 0.5.

The stress–strain curve of SBR with *D*_c_ of 8.0 at γ_0_ = 0.5 is extracted to observe the phenomenon of strain lagging behind stress. Figure 3 presents the stress–strain curve, which shows the strain curve is constantly delayed behind the stress curve by a constant time *δ*_t_ of 20 fs. Notably, the stress curve’s first two peaks are higher than the others. The phenomenon is predominantly attributed to the hindered movement of the entangled polymer chains by an external force, i.e., the shear stress. The interference results in relatively high viscosity and stress, leading to slightly higher peak values for the first two peaks. The originally entangled polymer chains’ entanglement points are released with the continued shearing, reducing the viscosity and leading to the gradual stabilization of the stress curve’s peak values.

**Figure 4:**
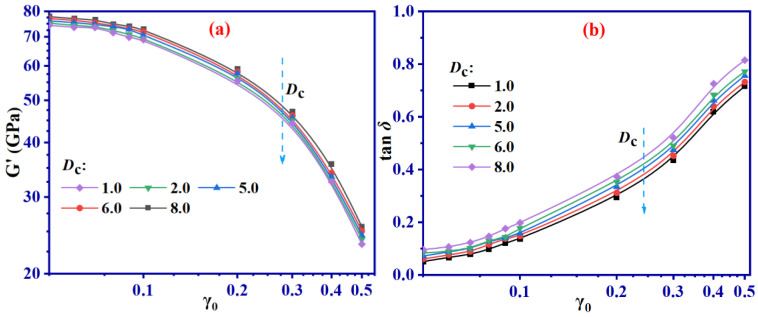
*G*′ (**a**) and tan*δ* (**b**) of SBR with different *D*_c_ under γ_0_ of 0.05–0.5.

The *G*′ and tan*δ* at different γ_0_ are presented in Figure 4.

Figure 4 illustrates that SBR with varying *D*_c_ manifests conspicuous Payne effects under varying γ_0_. Specifically, the *G*′ experiences a drastic decline with the augmentation of γ_0_, as depicted in Figure 4a. Additionally, Figure 4a shows that the *G*′ escalates with the intensification of *D*_c_. Figure 4b indicates that the tan*δ* of different *D*_c_ displays an increasing tendency as γ_0_ increases. To explore the formation mechanism of Payne effects and the *D*_c_ impact on dynamic performance during the shear process, detailed analysis from both a static and dynamic outlook are operated.

#### 2.2.1. Static Equilibrium Process

**Figure 5:**
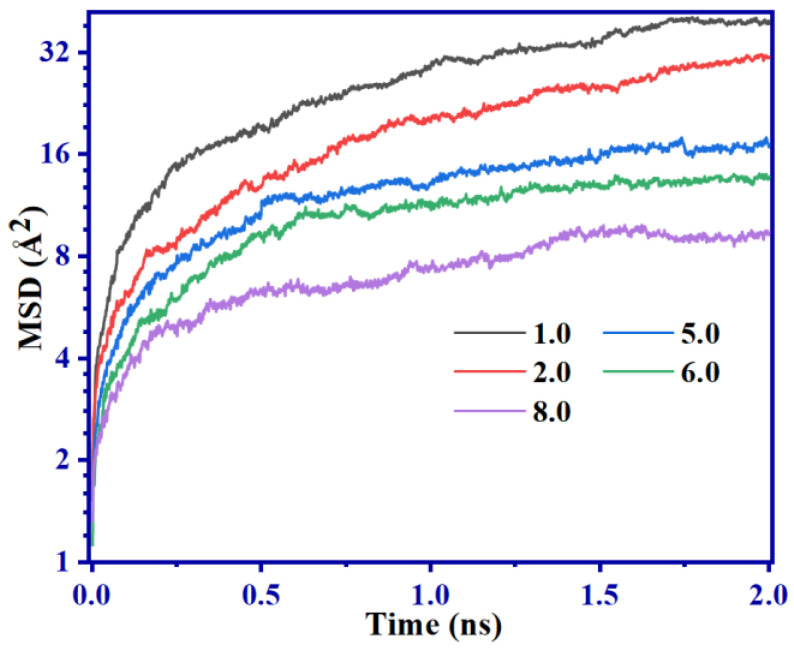
MSD function with time of 2 ns NPT balance in SBR systems with different *D*_c_.

**Figure 6:**
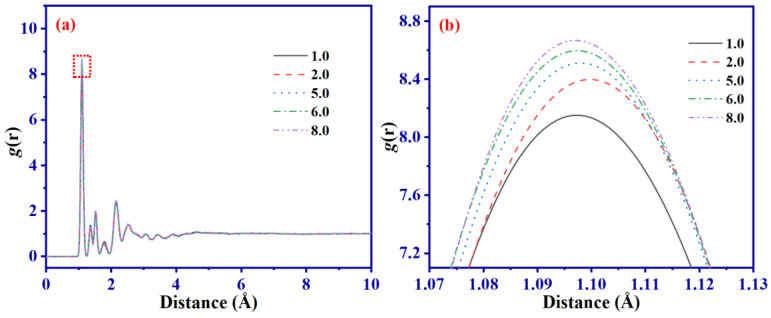
RDF (**a**) and local magnification at the red box in (**a**,**b**) at NPT equilibrium for SBR systems with different *D*_c_.

MSD and RDF of C atoms on the main chain are extracted from *D*_c_ systems with differing properties at equilibrium at the NPT for a period of 2 ns and presented in Figure 5 and Figure 6. The purpose of this extraction is to investigate the impact of movement of molecular chains and arrangement of atomic components on mechanical characteristics.

Observations from Figure 5 reveal that the MSD exhibits a decreasing trend with an increasing *D*_c_. Upon attaining equilibrium, the MSD of *D*_c_ = 1.0 SBR system is roughly 6 times more considerable than that of the *D*_c_ = 8.0 system. This is due to the abundance of C-S-C bonds, which progressively limit the movement of molecular chains, restricting their motion range and consequently their rigidity. This phenomenon, in turn, explains the increasing trend in *G*′ with *D*_c_ increment, as illustrated in Figure 4a.

The RDF depicted in Figure 6 reveals an increased density of C atoms present on the SBR main chain with an increment of *D*_c_. Specifically, for the *D*_c_ = 8.0 system, this density level is observed to be 10% higher than for the *D*_c_ = 1.0 counterpart. The reason for this occurrence is primarily attributed to the presence of more C-S-C bonds that support a tighter coupling between the SBR chains, as depicted in Figure 5.

#### 2.2.2. Microscopic Mechanism of Payne Effect

**Figure 7:**
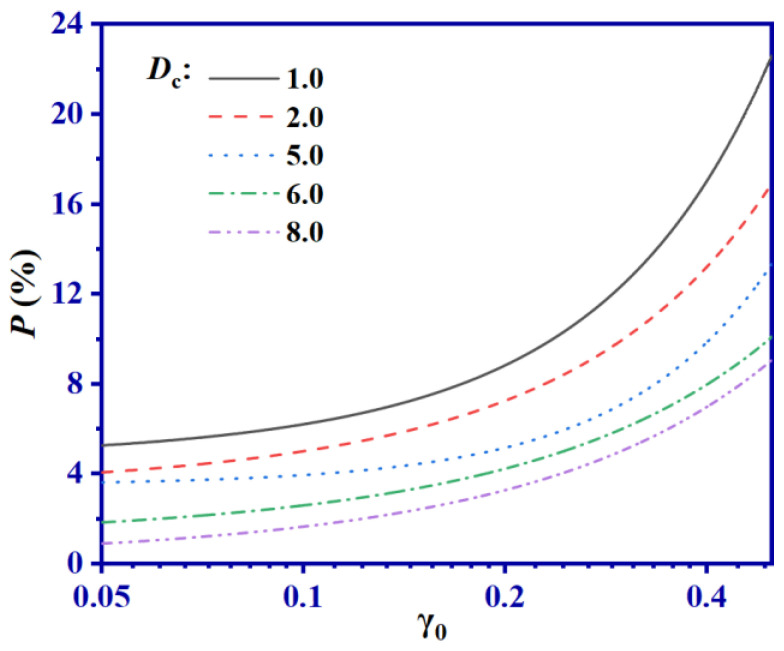
Relationship between *P* and γ_0_ in SBR systems with different *D*_c_.

In this work, we sought to examine the influence of cross-links on the shearing process by calculating the rate of broken cross-linking bonds, or *P*, after10 cycles of shearing. The calculation is performed as follows. If the length of a C-S bond linked with an S atom exceeded 1.8 Å, it is designated as “broken” and considered unrepairable during the shearing process. *P* is calculated from the ratio of broken cross-links (*N*_b_) to the total number of cross-linking bonds (*N*_s_), as given by *P* = *N*_b_/*N*_s_, where the subscript “b” represents “broken”. We present the relationship between *P* and γ_0_ for various SBR systems with differing *D*_c_ values in Figure 7.

**Figure 8:**
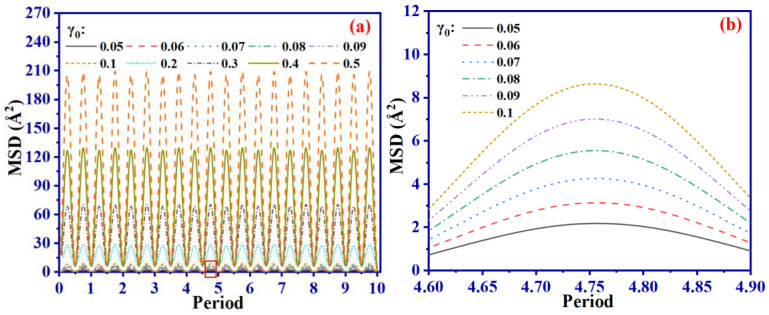

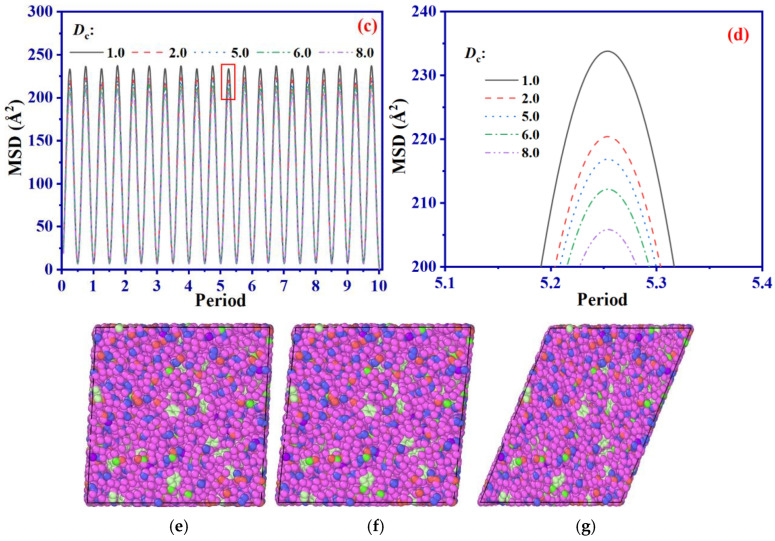
The variation function of MSD of SBR system with *D*_c_ = 8.0 at different γ_0_ (**a**) and variation function of MSD at different *D*_c_ at γ_0_ = 0.5 (**c**) with shear period; (**b**) is the local amplification at the red box in (**a**,**d**) and the local amplification at the red box in (**c**,**e**–**g**) are snapshots of the SBR system at shear strain limit with *D*_c_ = 8.0 at γ_0_ = 0.05, 0.1 and 0.5, respectively.

By analyzing Figure 7, it is evident that an increase in γ_0_ value leads to a corresponding increase in *P*. Larger γ_0_ values in a vulcanized SBR system cause the breakage of C-S-C bonds, which increases as γ_0_ increases. When γ_0_ exceeds 0.1, all SBR systems demonstrate a sharp rise in *P* value, consistent with the observation in Figure 4a. Therefore, it can be concluded that larger values of γ_0_ lead to a sudden increase in the number of broken cross-linking bonds, resulting in the occurrence of Payne effects during the shearing process, affecting the mechanical properties of the SBR system. Furthermore, the *P* value decreases as *D*_c_ increases. This implies that higher cross-linked densities exhibit lower *P* values after shearing. A plausible explanation for this observation is that larger *D*_c_ values can enhance the overall mechanical performance of SBR. During the shearing process, systems with lower *D*_c_ values tend to break more easily than those with higher *D*_c_ values, resulting in lower *P* values. The breakage of cross-linking bonds during the shearing process is mainly related to the movement of molecular chains. Therefore, taking the system with *D*_c_ = 8.0 as an example, the MSD during the shearing process is extracted under different values of γ_0_, as shown in Figure 8. In addition, Figure 8 also includes the MSD of different SBR systems with γ_0_ = 0.5 and varying *D*_c_.

It can be observed in Figure 8a that MSD is approximately 7.0 at γ_0_ = 0.09, but it significantly increases when γ_0_ exceeds 0.1, as seen in Figure 8e–g. For example, when γ_0_ = 0.5, MSD reaches approximately 230, which is 33 times that of γ_0_ = 0.09. Higher γ_0_ values move the system away from equilibrium, increasing the probability of cross-linking bond breakage and potentially decreasing mechanical performance, as explained by the significant decline in *G*′ shown in Figure 4a when γ_0_ is greater than 0.1. Furthermore, MSD decreases as *D*_c_ increases under the same γ_0_ value, as shown in Figure 8c,d. This is primarily due to the partial restriction of molecular chain movement by the formation of the cross-linking network, consequently slowing the occurrence of bond breakage. Hence, when γ_0_ remains the same, an increase in *D*_c_ results in a higher *G*′.

**Figure 9:**
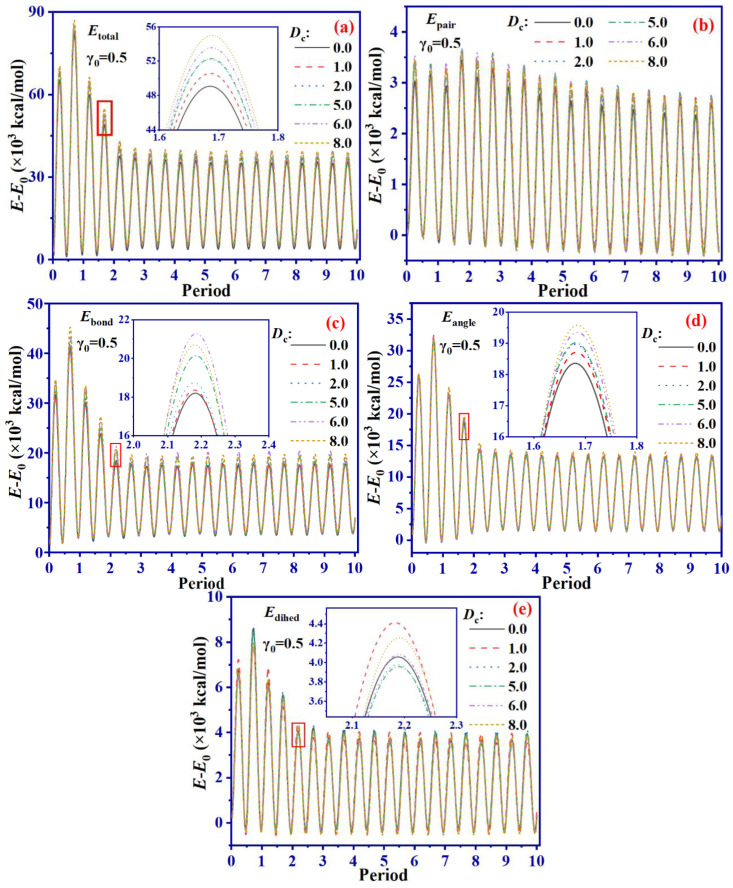
The change function of *E*_total_ (**a**), *E*_pair_ (**b**), *E*_bond_ (**c**), *E*_angle_ (**d**) and *E*_dihed_ (**e**) with shear period of different *D*_c_ SBR systems at γ_0_ = 0.5.

To analyze the energy variation in SBR systems during the shearing process, we calculate both the non-bonding energy (*E*_pair_, which describes the repulsive or attractive forces between two non-bonded atoms and can be calculated by the sum of the van der Waals energy and Coulomb energy) as well as the bonding energy, which included bond energy (*E*_bond_, which represents the stretching energy of a bond and characterizes the energy change resulting from the movement of each chemical bond in the direction of the molecular axis), angle energy (*E*_angle_, which represents the bending energy of a bond angle, caused by changes to the bond angle) and dihedral energy (*E*_dihed_, which represents the energy change caused by the rotation of a single bond and resulting in molecular skeleton distortion) over the period of shearing. Initially, we extracted *E*_total_, *E*_pair_, *E*_bond_, *E*_angle_, and *E*_dihed_ of SBR systems with different values of *D*_c_ during the shearing process at γ_0_ = 0.5, as depicted in Figure 9 (where *E*_total_ = *E*_pair_ + *E*_bond_ + *E*_angle_ + *E*_dihed_). We also present the energy variation of a non-cross-linking system SBR system (*D*_c_ = 0) during the shearing process in Figure 9 for comparing the specific effects of different cross-linking densities on energy changes. We consider the energy difference (*E*-*E*_0_) at each moment of the shearing process relative to the system’s initial energy [25] to facilitate analysis.

It is obvious in Figure 9 that the energy of the various *D*_c_ systems fluctuates sinusoidally during shear and gradually decreases. This behavior can be adequately explained by combining Figure 3b which results from the peak stress increase. Additionally, an increase in *D*_c_ leads to a rise in *E*_total_, which increases by approximately 12% for *D*_c_ = 8.0 compared to the non-cross-linking system. This can mainly be attributed to the fact that the higher *D*_c_ system has more cross-linking bonds, thus increasing the *E*_total_ of the system. Furthermore, it can be observed from Figure 9b that *E*_pair_ does not significantly change as *D*_c_ increases. This is due to SBR chains being mainly cross-linked by C-S-C bonds, meaning that interatomic interaction forces do not show significant differences.

In contrast to *E*_pair_, both *E*_bond_ and *E*_angle_ corresponding to Figure 9c,d exhibit correlation with *D*_c_. The peak values of *E*_bond_ and *E*_angle_ increase and show an increase of 19.4% and 6.5%, respectively as *D*_c_ increases, when compared to the non-cross-linking system. Figure 9e shows that there is no significant correlation with respect to changes in *D*_c_ for *E*_dihed_.

**Figure 10:**
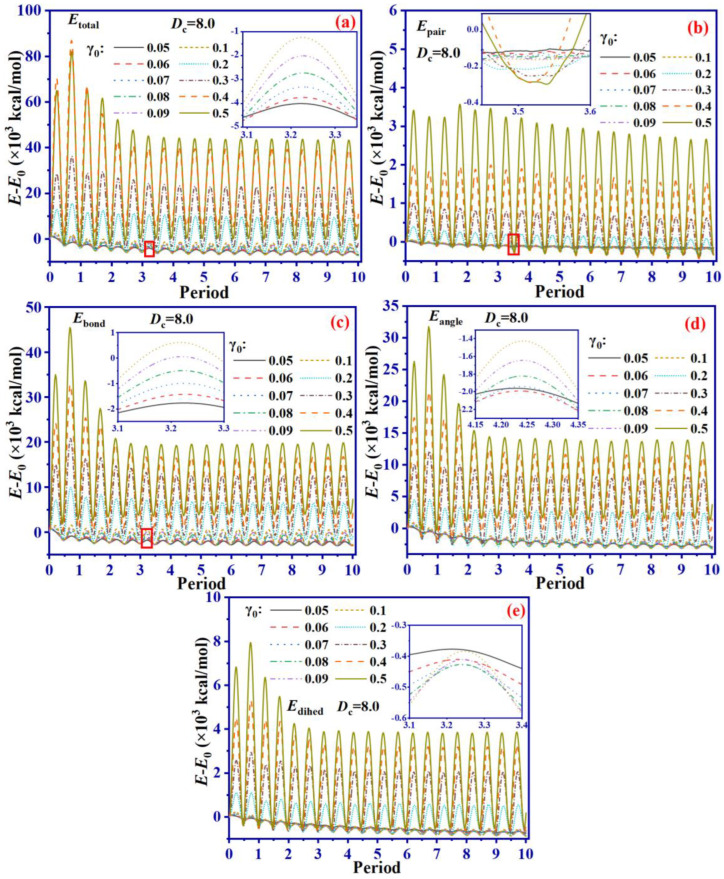
*E*_total_ (**a**), *E*_pair_ (**b**), *E*_bond_ (**c**), *E*_angle_ (**d**) and *E*_dihed_ (**e**) of SBR system with *D*_c_ 8.0 at different γ_0_.

To sum up, from an energy perspective, changing *D*_c_ mainly affects the stretching of bonds and the rotation of bond angles during the shearing process under the same γ_0_ conditions. As for the rotation of bond angles, *D*_c_ has a more significant impact on bond stretching in the cross-linking SBR system. In order to investigate the impact of different γ_0_ on the energy during the shearing process, we extract the energy variation of the system with *D*_c_ = 8.0 under different γ_0_, as illustrated in Figure 10.

According to Figure 10a, the *E*_total_ displays periodic variation with two peaks during each shear cycle, corresponding to the maximal and minimal strains of the action, respectively. As the parameter γ_0_ increases, the peaks of *E*_total_ also increase. This finding has been previously discussed. The process of shearing depletes *E*_total_ gradually, primarily due to the viscoelasticity of SBR absorbing energy through overcoming the frictional force of molecular chains [26]. Consequently, the energy reduction rate tends to be slower under lower strain conditions, when molecular movement gets more limited, and resulting in lower frictional resistance between chains, causing slower dissipation of energy. Figure 10c,d report alterations in *E*_bond_ and *E*_angle_ during shearing. The shift trends of these values lead to the conclusion that the energy change during shearing is mainly driven by bond stretching and angle variation. The sum of *E*_bond_ and *E*_angle_ accounts for more than half of *E*_total_, making these the primary contributors to the shearing status of the system. During the shearing process, stretching bonds and rotating bond angles mainly determine the deformation mode of the system. *E*_bond_ tends to reach equilibrium at 20,000 kcal/mol and *E*_angle_ at 14,000 kcal/mol. These findings suggest that bond stretching has a superior role concerning bond angles rotation in the shearing process. Dihedral angle distortion plays a relatively minor part in contrast, Figure 10e shows that *E*_dihed_ exhibits periodic variation and increases with γ_0_, while gradually decreasing with an increase in shearing cycle. When γ_0_ = 0.05, *E*_dihed_ is around −400 kcal/mol and when γ_0_ = 0.5, *E*_dihed_ is around 4000 kcal/mol. The principal reason for the raised value of *E*_dihed_ is the increase in the distance between the molecular chains and their initial position due to a larger γ_0_.

**Figure 11:**
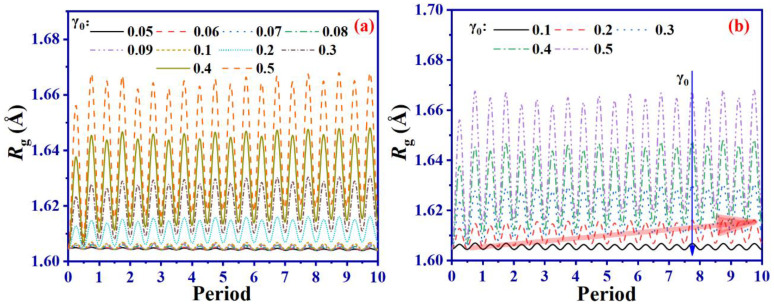

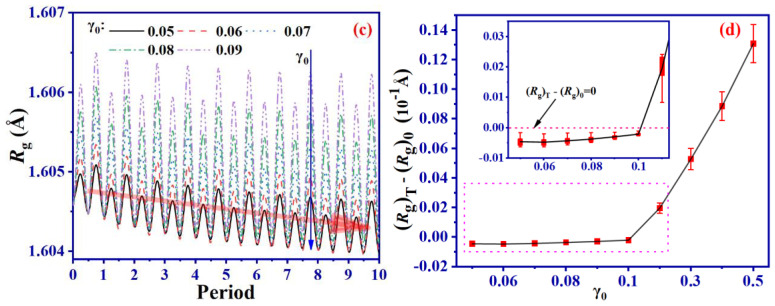
*R*_g_ of the system under different γ_0_ (**a**–**c**) varies with the shear period and the difference between the *R*_g_ and the initial *R*_g_ ((*R*_g_)_T_-(*R*_g_)_0_) after 10 shear periods under different γ_0_ (**d**).

To study the changes in molecular chain conformation during the shearing process, we extract the mean square radius of gyration (*R*_g_) of the molecular chain at *D*_c_ = 8.0 under different γ_0_, calculated by Equation (1) and shown in Figure 11.

(1) $$R_{g}^{2}=\frac{1}{M}\sum_{i}m_{i}{\left(r_{i}-r_{cm}\right)}^{2}$$

where *M* is the total of the model, *r*_cm_ is the center-of-mass position, and the sum is over all atoms in model.

The *T*_g_ is the primary mechanism for examining the impact of molecular chain flexibility on dynamic mechanical properties. A larger *R*_g_ indicates poorer flexibility and molecular chains with poorer flexibility correspond to higher *T*_g_ and poorer dynamic mechanical performance at the same temperature [27,28].

From Figure 11a–c, it is evident that the *R*_g_ of the molecular chain undergoes periodic variation during the shearing process, with two peaks in one shear cycle, corresponding to the two displacement limits during the shearing process. With the increase in parameter γ_0_, the *R*_g_ peaks increase, implying a decreased flexibility of molecular chains during shearing, ultimately leading to diminished dynamic mechanical properties of vulcanized SBR.

The results from Figure 11 reveal that *R*_g_ remains almost unchanged, i.e., independent of the γ_0_ when γ_0_ is less than 0.1 (Figure 11c). On the contrary, *R*_g_ demonstrates a noticeable upward pattern with γ_0_, as determined by the difference between the initial *R*_g_ and the value after 10 shearing cycles, when γ_0_ exceeds 0.1 (Figure 11b,d). As the value of γ_0_ increases, the molecular chains become progressively inflexible, causing a significant increase in *R*_g_ and a reduction in the mechanical properties. This trend is further demonstrated in Figure 4a, which illustrates the *G*′ of SBR, showing no obvious reduction when γ_0_ falls below 0.1. However, when γ_0_ reaches and surpasses 0.1, the *G*′ of SBR dwindles considerably.

## 3. Materials and Methods

### 3.1. Verification of Simulation Correctness

The cross-linking densities (mol/mL) corresponding to each *D*_c_ in MD model obtained via calculation are presented in Table 3. The results of cross-linking densities obtained by experiments are also shown in Table 3.

A model of vulcanized SBR is established by changing the cross-linking density (*D*_c_) according to the method in Equation (2). *D*_c_ values of 1.0, 2.0, 5.0, 6.0, and 8.0 are assigned for the model.

(2) Dc=Ns/Mc

where *N*s is the number of cross-link bonds and *M*c is the number of single chains.

The cross-linking criteria are defined as follows. If the distance between two carbon atoms (C) on the main chain falls within the range of 1.6 to 3.45 Å, a sulfur atom (S) is added to the geometric center of the two C atoms and a C-S-C bond is formed [29]. Additionally, since every two C atoms on a single chain can create a C-S-C bond, the phenomenon of self-cross-linking is considered in the actual cross-linking process [30]. Once a C atom on the main chain establishes a cross-link bond, it can no longer bind to other S atoms. To implement the complete cross-linking process, this study utilized *Perl* scripts inside Materials Studio software.

The *pcff* force field is utilized to describe the intermolecular and intramolecular interactions in SBR. Equation (3) depicts the formula of the *pcff* force field.

(3) $$\begin{array}{l} E_{pot}=\sum_{b}\left[k_{2}{\left(b-b_{0}\right)}^{2}+k_{3}{\left(b-b_{0}\right)}^{2}+k_{4}{\left(b-b_{0}\right)}^{2}\right]\\ +\sum_{\theta }\left[H_{2}{\left(\theta -\theta_{0}\right)}^{2}+H_{3}{\left(\theta -\theta_{0}\right)}^{3}+H_{4}{\left(\theta -\theta_{0}\right)}^{4}\right]\\ +\sum_{\varphi }\left[V_{1}(1-\cos\varphi )+V_{2}(1-\cos2\varphi )+V_{3}(1-\cos3\varphi )\right]+\sum_{\chi }K_{\chi }\chi^{2}\\ +\sum_{b,b^{\prime }}F_{bb}(b-b_{0})(b^{\prime }-b^{\prime }{}_{0})+\sum_{b,\theta }F_{b\theta }(b-b_{0})(\theta -\theta_{0})+\sum_{\theta^{\prime }\theta^{\prime }}F_{\theta \theta^{\prime }}(\theta -\theta^{\prime })(\theta -\theta^{\prime })\\ +\sum_{b,\varphi }\left(b-b_{0})(V\cos\varphi +V_{2}\cos2\varphi +V_{3}\cos3\varphi \right)\\ +\sum_{\theta ,\varphi }\left(\theta -\theta_{0})(V\cos\varphi +V_{2}\cos2\varphi +V_{3}\cos3\varphi \right)\\ +\sum_{b,\theta ,\varphi }F_{b\theta \varphi }(b-b_{0})(\theta -\theta_{0})\cos\varphi \\ +\sum_{i,j}\frac{q_{i}q_{j}}{\varepsilon r_{ij}}\\ +\sum_{i,j}\left[\frac{A_{ij}}{r_{ij}{}^{9}}-\frac{Bi_{j}}{r_{ij}{}^{6}}\right] \end{array}$$

**Figure 12:**
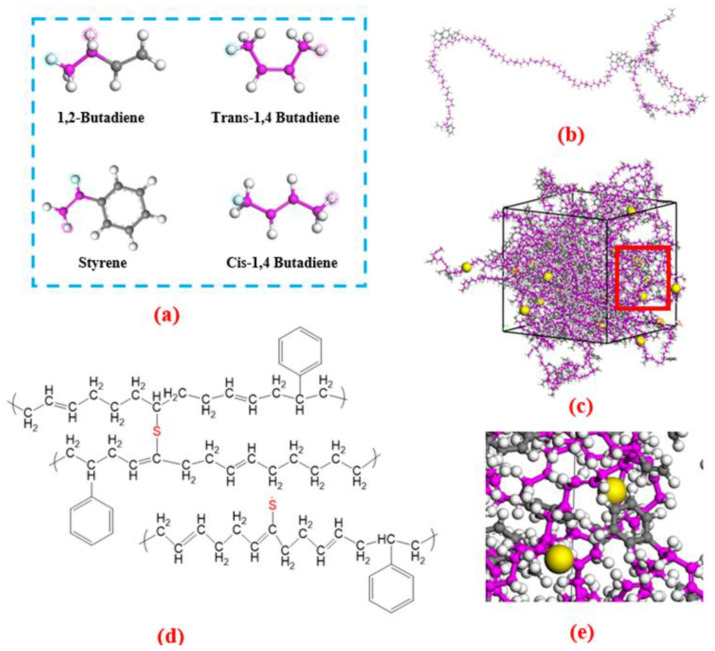
The modeling details of the cross-linking include four monomers in SBR chain (**a**), single chain containing 60 monomers (**b**), the model after cross-linking (**c**), molecular formula (**d**) and the local amplification (**e**) at the red box in (**c**). In (**c**,**e**), the magenta beads are C atoms on the main chain, the gray beads are C atoms of the benzene ring, the white beads are the H atoms and the yellow beads are the S atoms.

Van der Waals forces are calculated by truncated Lennard-Jones (L-J) 9-6 equation, illustrated in Equation (4).

(4) $$V_{LJ}(r)=\left\{\begin{array}{ll} 4\varepsilon {\left(\frac{\sigma }{r}\right)}^{9}-{\left(\frac{\sigma }{r}\right)}^{6} & \;(r\leq r_{c})\\ 0 & \left(r>r_{c}\right) \end{array}\right.$$

where *ε* is the bond energy at the equilibrium position where the force *F*(*r*) = 0, *σ* is the collision diameter, *r*_c_ is the truncation radius of 9.5 Å, and *r* refers to the interatomic distance. Figure 12 presents the molecular formula, process and results of cross-linking.

After the completion of cross-linking, we perform relaxation annealing on the model. The specific method is as follows. Firstly, a 2 ns NPT relaxation is carried out at 300 K and 1 atm, then the model is heated to 600 K and then cooled to 300 K in NVT ensembles to simulate the annealing process. This process lasted for 4 ns and is repeated 4 times. Finally, a 2 ns NPT relaxation is performed at 300 K and 1 atm, during which the radial distribution function (RDF) and mean square displacement (MSD) of the system are extracted. The obtained model is used for studying the dynamic shear process.

This study investigated the dynamic mechanical properties of SBR with varying density of cross-linking (*D*_c_) through cyclic shear deformation of simulated box. The shear deformation is performed in the *XY* plane by moving the simulation box along the ±*X* directions with a fixed shear rate of 0.01 ps^−1^. The shear strain amplitude (γ_0_) varied from 0.05 to 0.5 and the system underwent 10 complete shear cycles. The changes over time in shear stress (*σ*) and strain (*γ*) are expressed using Equations (5) and (6). Consequently, the storage modulus (*G*′) and loss factor (tan*δ*) of SBR with varying *D*_c_ are obtained through Equation (7).

(5) $$\sigma =\sigma_{0}\sin\;(\omega \;t+\delta )$$

(6) $$\gamma =\gamma_{0}\sin(\omega \;t)$$

(7) $$G^{\prime }=\frac{\sigma_{0}}{\gamma_{0}}\cos(\delta )\;\;\tan\delta =G^{\prime \prime }/G\prime$$

An Anderson thermostat and Berendsen barostat are employed to control temperature and pressure, respectively, for all simulation in this study, while the velocity Verlet algorithm is used for kinetic integration. Molecular dynamics simulations are performed using the LAMMPS package on a 24-core supercomputer [31]. The visualizing results are analyzed through Open Visualization Tool (OVITO) software [32].

### 3.2. Experimental Methods

The amounts of sulfur addition in vulcanized SBR are determined based on the S atomic mass fraction (phr) in different *D*_c_ models. The determined amounts are 1.04, 1.99, 5.03, 6.29, and 8.28, respectively. The mixed SBR is obtained by adding compounding agents to pure SBR. Table 4 illustrates the content of each component.

Pure SBR is premixed for 2 min on an open mill with a diameter of Φ152.4 mm. Subsequently, ZnO, SA, accelerant DZ, accelerant TT, and insoluble sulfur (S) are added in sequence to produce mixed SBR. The mixed rubber is allowed to stand for 24 h before testing the vulcanization properties and recording *T*_90_ using a non-rotor vulcanizer produced by Gotech. Vulcanizations of each mixed rubber are carried out at 150 °C to create a 2 mm vulcanized SBR sheet with a temperature of 150 °C, and the time of vulcanization is calculated based on *T*_90_ value.

#### 3.2.1. Cross-Linking Density Test

After allowing the vulcanized SBR to stand for 24 h, the cross-linking density analyzer with the model XLDS-15 is used to test the cross-linking density by the method of nuclear magnetic resonance (NMR). The SBR samples are tested under specific conditions of 90 °C for the temperature, 0.35 T for the magnetic field intensity and 15 MHz for the resonance frequency. The cross-linking density is calculated by measuring the relaxation time (*T*_2_) of the SBR samples with varying sulfur contents. The same formulae are tested five times and the average values are adopted as the final result.

#### 3.2.2. DSC Test

The differential scanning calorimeter model DSC 200 F3 is utilized to quantify the glass transition temperature (*T*_g_) of the vulcanized SBR. The sample weighing 8–10 mg is then loaded into the calorimeter and heated to 100 °C for 5 min to eliminate the thermal history. Next, the temperature is lowered to −75 °C and held for five minutes before being heated back to 100 °C. The test parameters include both the heating and cooling rates of 10 °C/min.

#### 3.2.3. RPA Test

The RPA2000 rubber processing analyzer (RPA) is employed for evaluation of the dynamic mechanical properties of each sample. Initially, the temperature is set to 150 °C and the samples are subsequently put into the testing equipment while recording the time taken. Upon reaching the corresponding *T*_90_ to simulate the vulcanization process, the instruments’ temperature is decreased to 20 °C, after which the samples undergo a shearing test, the test frequency is set at 10 Hz and strain range is 0–55%.

### 3.3. Experimental Validation

**Figure 13:**
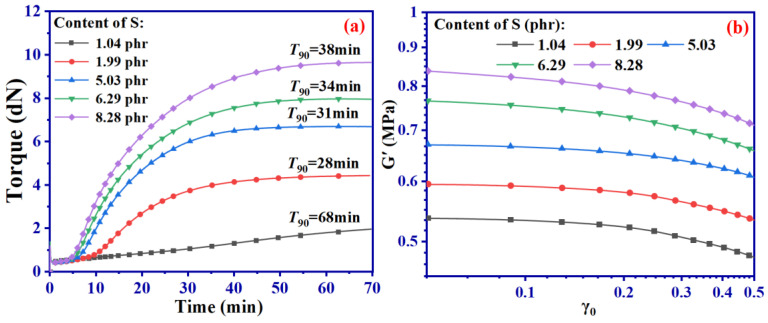
Vulcanization curve of SBR with different contents of S (**a**) and the variation of *G*′ with γ_0_ tested by RPA (**b**).

Figure 13 shows the vulcanization curve of SBR with different contents of S and the variation of *G*′ with γ_0_ tested by RPA.

Figure 13 shows an increase in the Payne effect in all groups of SBR with an increase in γ_0_. As observed from combining these findings with the results presented in Figure 5, it is apparent that higher S content enhances the deformation resistance of SBR, which is demonstrated by a noticeable upward trend in *G*′ (Figure 13). Furthermore, as γ_0_ exceeds 0.1, *G*′ reduction increases in all groups of SBR.

However, the experimental and simulated results presented in Figure 4 and Figure 13 display a difference of five orders of magnitude in their *G*′ values; nevertheless, the overall trends remain consistent. This means that both experimental and simulated outcomes confirm that vulcanized SBR exhibits a distinct Payne effect. The significant difference in magnitude is due to the scale effect of MD simulation. Consequently, the experimental results can provide more reliable evidence to support our simulation approach.

## 4. Conclusions

In this study, molecular dynamics (MD) simulations are conducted to study the SBR models with different *D*_c_, which are then subjected to shear deformation experiments for verification. The main conclusions of this study are as follows.

1. As *D*_c_ increases, the *T*_g_ of the SBR also increases. When *D*_c_ = 8.0, *T*_g_ is 243.84 K, which is 5.45% higher than the *T*_g_ of 231.23 K obtained for *D*_c_ = 1.0. The maximum difference between experimental and simulation results is 5.65%.
2. An increase in *D*_c_ limits the movement of molecular chains. When *D*_c_ = 1.0, MSD is about six times that of *D*_c_ = 8.0. RDF analysis proves that the larger *D*_c_ makes the atomic distribution in the system more compact, which is one of the reasons for higher *D*_c_ having a higher *G*′.
3. Dynamic shear deformation dissipates about 48% of the system’s energy through bond stretching and 37% through bond angle rotation. Changes in *D*_c_ and γ_0_ all have a significant impact on bond stretching, but little impact on bond angle rotation. In addition, the dihedral rotations have the lowest contributions in the Payne effect.
4. SBR with different *D*_c_ show significant Payne effect under dynamic shear deformation. This is primarily due to the deviation of molecular chains from the initial position, leading to bond breaking and reduced flexibility of molecular chains. When γ_0_ > 0.1, the difference between (*R*_g_)_0_ and (*R*_g_)_T_ corresponding to each shear period increases significantly.
5. In summary, this study provides theoretical guidance and methodological insights for future research on sulfurized SBR.

## Figures and Tables

**Table 1:** Cross-linking densities (mol/mL) in MD model and experiment results.

*D*_c_	1.0	2.0	5.0	6.0	8.0
Content of S (phr)	1.04	1.99	5.03	6.29	8.28
Cross-link density by MD (10^−4^ mol/mL)	1.208	1.48	1.681	2.117	2.269
Cross-link density by experiment (10^−4^ mol/mL)	1.194	1.464	1.659	2.088	2.247

**Table 2:** *T*_g_ and relative error of SBR obtained by DSC and MD simulation.

Content of S (phr)	*T*_g_ by MD Simulation (K)	*T*_g_ by Experiment (K)	Relative Deviation (%)
1.04	231.23	219.17	5.5
1.99	235.79	223.13	5.65
5.03	238.13	227.47	4.69
6.29	241.74	232.02	4.19
8.28	243.84	236.10	3.28

**Table 3:** Monomer components in single-chain SBR.

Monomer	Styrene	1,4 Butadiene	Cis-1,2 Butadiene	Trans-1,2 Butadiene
Contents (wt. %)	23.5	14	12.5	50

**Table 4:** The content of each component in mixed SBR.

Components	SBR	SA	Accelerant DZ	Accelerant TT	ZnO	S
Contents (phr)	100	1	1	0.1	4.0	Variable

## Data Availability

The data that support the findings of this study are available on request from the corresponding author.
